# Progressive Temporal-Spatial-Semantic Analysis of Driving Anomaly Detection and Recounting

**DOI:** 10.3390/s19235098

**Published:** 2019-11-21

**Authors:** Rixing Zhu, Jianwu Fang, Hongke Xu, Jianru Xue

**Affiliations:** 1 School of Electronic and Control Engineering, Chang’an University, Xi’an 710064, China; rixingzhu@chd.edu.cn (R.Z.); xuhongke@chd.edu.cn (H.X.); 2 Institute of Artificial Intelligence and Robotics (IAIR), Xi’an Jiaotong University, Xi’an 710049, China; jrxue@mail.xjtu.edu.cn

**Keywords:** driving anomaly, temporal-spatial-semantic analysis, isolation forest, semantic causal relation

## Abstract

For analyzing the traffic anomaly within dashcam videos from the perspective of ego-vehicles, the agent should spatial-temporally localize the abnormal occasion and regions and give a semantically recounting of what happened. Most existing formulations concentrate on the former spatial-temporal aspect and mainly approach this goal by training normal pattern classifiers/regressors/dictionaries with large-scale availably labeled data. However, anomalies are context-related, and it is difficult to distinguish the margin of abnormal and normal clearly. This paper proposes a progressive unsupervised driving anomaly detection and recounting (D&R) framework. The highlights are three-fold: (1) We formulate driving anomaly D&R as a temporal-spatial-semantic (TSS) model, which achieves a coarse-to-fine focusing and generates convincing driving anomaly D&R. (2) This work contributes an unsupervised D&R without any training data while performing an effective performance. (3) We novelly introduce the traffic saliency, isolation forest, visual semantic causal relations of driving scene to effectively construct the TSS model. Extensive experiments on a driving anomaly dataset with 106 video clips (temporal-spatial-semantically labeled carefully by ourselves) demonstrate superior performance over existing techniques.

## 1. Introduction

The main goal of this paper is to detect and recount (D&R) the driving anomaly recorded by dashcam videos in the perspective of ego-vehicle (driving vehicle itself). “Detect” means to localize the anomaly occasion (reflected by video frame interval) and local anomaly region (target region of the anomaly). “Recount” aims to fulfill a reasonable semantic explanation of driving anomaly as much as possible, such as “what hits what”. The “Detect and Recount” framework is more useful for the automatic driving anomaly explanation. Analyzing the process of driving anomaly may pave the anticipation of accident and disclose the potential state-chain from a driving anomaly to an accident.

Most related works to this paper mainly concentrate on the anomaly detection [1,2,3,4,5,6,7,8,9,10] in surveillance. Most of them are devoted to spatial (pixel-level) and temporal (frame-level) localization of anomalies accurately, and adopt large-scale normal data to train normal discriminators (classifiers, regressors, or dictionaries) for detecting the abnormal patterns (features extracted) deviating from the trained discriminators. However, spatial-temporal localization is difficult because of the ambiguous margin of abnormal and normal situation. Actually, this is the same for our driving anomaly while needing a further semantic explanation for the evolution process. Because the anomaly is context-related [11] and difficult to tag, this paper presents an unsupervised driving anomaly D&R, and the proposed temporal-spatial-semantic (TSS) model can fulfill a coarse-to-fine focusing of driving anomaly from temporal-spatial to semantic, and achieve a convincing D&R.

**Figure 1:**
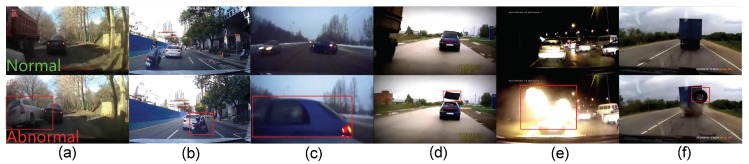
Typical driving anomalies of (**a**) vehicle-to-vehicle crash, (**b**) motorbike-to-vehicle crash, (**c**) vehicle crossing, (**d**) vehicle roof throwing, (**e**) vehicle catching fire, and (**f**) falling tire.

Different from traditional anomaly detection in surveillance, driving anomalies generally have more explicit context, but more complicated motion, challenging situations and weather conditions, as shown in Figure 1, which may cause hazards for the ego-vehicle or a driving accident. Although most of the driving anomalies are related to the moveable targets (pedestrians or vehicles), which cannot cover all causations in the driving anomaly, e.g., the falling tire shown in Figure 1f.

**Figure 2:**
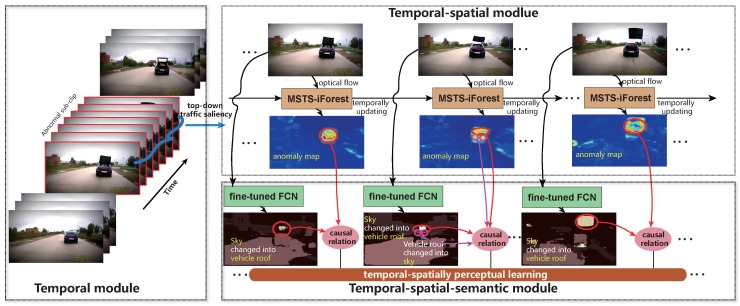
The temporal-spatial-semantic (TSS) model for driving anomaly detection and recounting.

The coarse-to-fine TSS model aims to contain most of the anomaly elements in the beginning temporal module, and focalizes the anomaly elements by following spatial and semantic modules. The flowchart is illustrated in Figure 2 and expressed as follows.

Temporal module. In the temporal module, we novelly introduce the top-down traffic saliency to represent the eye fixation variation caused by varying driving scenes. The underlying meaning is that traffic saliency reflects the driving context promisingly [12,13,14], which tells where the drivers look in different scenarios, and anomalies usually generate sudden eye fixation variation. We extract the frames with sudden eye fixation variation to provide temporal anomaly candidates.

Temporal-spatial module. Temporal-spatial module aims to detect the local anomaly regions within the extracted temporal window. In this step, we introduce the isolation forest (iForest) for an unsupervised abnormal point separation. Differently, we contribute a multi-scale temporal-spatial isolation forest (MSTS-iForest), and enforce the optical flow to represent the element behavior. MSTS-iForest can restrict the estimation error of the optical flow, and make the anomaly region more obvious. Different from surveillance scenarios, motion anomaly is the primary in this work.

Temporal-spatial-semantic module. Temporal-spatial-semantic module focuses on associating the detected local anomaly regions with generic semantic knowledge oriented by the special driving environment, correlating to the elements on the road scene (e.g., person, cyclist, motorbike, vehicle, road, sidewalk, etc.). For embedding the generic semantic knowledge, this work encodes the video frame by fully convolutional networks (FCN) [15] fine-tuned by Cityscapes’ semantic segmentation benchmarks [16], and generates the semantic class possibility of each pixel. Then, we recount the semantic variation of the pixels within the detected anomaly regions and determine the potential causal relations of those anomaly semantic variations.

Based on the aforementioned content, the contributions are as follows:

- This paper contributes an unsupervised driving anomaly detection and recounting (D&R) while performing an effective performance, which does not need any training data.
- A temporal-spatial-semantic (TSS) model is constructed to fulfill a coarse-to-fine focusing of the driving anomaly D&R. For each module, we design the procedure meticulously to find temporal, spatial, semantic cues for driving anomaly D&R.
- We validated the superiority of the proposed method by a dataset containing 106 video clips (100 frames/clip) temporal-spatial-semantically labeled by ourselves carefully.

## 2. Related Works

Detecting driving anomalies is of great significance for promoting driving safety and reducing risk. The development of an on-board monitoring system has made it feasible to detect driving anomalies by multiple vehicle sensors [17,18,19], such as GPS, video, and 3D-LiDAR, etc. For ego-vehicles, anomaly detection plays a major component in video analysis [20,21]. Video anomaly is commonly defined as the target behavior which occurs rarely, inconsistent with pre-defined normal model/rules and context-deviated [4,11]. Many efforts are devoted to involve the model of the distribution of majority normal behavior, spatial, and temporal consistency/dependency of behaviors. It is worth noting that this is similar for generic anomaly detection in surveillance and driving anomaly detection [22]. Therefore, the approaches for anomaly detection can be categorized as normal behavior modeling and spatial-temporal consistency. For this work, we also review anomaly recounting.

Normal behavior modeling. For modeling the normal behavior, exploring the normal rules contained in the trajectories is a standard approach [23,24], which can capture the long-term semantics of objects while often failing to track accurately because of various disturbing factors, e.g., occlusion, fast motion, similar object surrounded, and so on. Hence, the alternatively recent approaches unitized the hand-craft low-level features (e.g., HOG, HOF, STIPs, etc.) extracted from 2D or 3D frame region(s). Commonly, these locally low-level features are feeded into various detectors trained by normal samples, such as distance-based [25], sparse-coding [1,26], domain-based (one class SVM) [27], probabilistic-based (e.g., mixture of probabilistic PCA (MPPCA) [28], and Gaussian process regressor [25]), Graph-based inference machines [29], and physical-inspired models [30]. Some recent models adopted the deep features or original images to learn autoencoders [2], expressive normal CNNs [3,31] or predictive RNNs [32,33,34], and minimized the reconstruction/expression/prediction error of the input samples. Most related to this paper, Chan et al. [32] proposed a dynamic-spatial-attention (DSA) recurrent neural network (RNN) for anticipating accidents in dashcam videos, where soft-attention was distributed to candidate objects and utilized a temporal dependency. Normal behavior modeling needs training data prepared, whereas it is difficult to mask the margin of abnormal and normal clearly.

Spatial and temporal consistency. Spatial-temporal consistency is mainly inspired by the co-occurrence of appearance or motion pattern in local spatial region and over temporal frames, and filters the local anomaly scores obtained by aforementioned normal discriminators. For example, Kratz and Nishino [5] involved the correlation of appearance and motion behavior with a state-variation matrix, and transferred the state by hidden Markov model (HMM). Basharat et al. [35] placed emphasis on the sequential evolution of tracklets and object scale variation between frames, and inferred the consistency by Gaussian mixture model (GMM). Spatial-temporal mixture of dynamic texture (MDT) [6] was adopted to build the appearance variation of local regions over frames. Gaussian process regression (GPR) [25] was also taken to smooth the local anomaly scores and obtain the global frame-level anomaly. Yuan et al. involved spatial-temporal context consistency of pedestrians to conduct the crowd anomaly [36], and had addressed the driving anomaly by motion consistency [37]. For representing the spatial-temporal consistency, some works embedded the high-level structure consistency for anomaly detection in videos, such as the feature grouping of individuals by manifold learning [38,39,40]. In addition, because of the great success of CNN or RNN approaches in many visual tasks, the most recent approaches approximated the spatial-temporal consistency by exploiting the dependency of the behaviors between frames, such as LSTM predictor [34] and sequential generator [41]. For instance, Liu et al. [41] leveraged a future frame prediction based framework for anomaly detection by generative adversarial networks (GANs).

Anomaly recounting. The main goal of anomaly recounting is to explain the semantic evidence in detecting anomaly. Different from the mostly investigated multi-media event recounting (MER) [42,43] focusing on the activity of all the individuals in the scene, anomaly recounting concentrates on the abnormal elements. For anomaly recounting, there is only one work established by Hinami et al. [44] incorporated the object, action, and attribute (e.g., color) together by a multi-task Fast RCNN, by which the environment-dependent knowledge was learned. However, as aforementioned, it is difficult to cover all the abnormal elements in driving environment.

## 3. Driving Anomaly Detection and Recounting

### 3.1. Problem Formulation

In this work, we want to contribute a human-like driving anomaly detection, i.e., temporal anomaly window determination, detect the spatial anomaly regions in the temporal anomaly window, and give a recounting for the temporal-spatial anomaly. The purpose is to find more details about the driving anomaly. Therefore, we fulfill a coarse-to-fine setting for this work.

Given a video clip $V=\{X_{1},X_{2},...,X_{t},...,X_{T}\}$ captured in a driving scenario, where $X_{t},t\in \left[1,T\right]$ denotes the $t^{th}$ frame in V, the driving anomaly detection problem in this paper can be formulated as an unsupervised search of *K* abnormal sub-clips $\{C_{1},...,C_{k},...,C_{K}\}\in V$, where abnormal regions truly exist in $C_{k}$, and are accurately localized. Here, we denote the frames in $C_{k}$ as ${\left\{X_{m}^{k}\right\}}_{m=1:M},M\ll T$. The recounting problem aims to express the semantic evolution process within $C_{k}$. To be clearer, we formulate driving detection and recounting by a temporal-spatial-semantic (TSS) model, which is a nest structure, i.e., (1) temporally searching *K* abnormal sub-clips $\left\{C_{1},...,C_{k},...,C_{K}\right\}$; (2) temporal-spatially localizing abnormal regions $A_{m}^{k}$ of each frame $X_{m}^{k}$ in abnormal sub-clip $C_{k}$, k=1,...,K; (3) temporal-spatial-semantically recounting the evolution process of $A_{m}^{k}$, m=1,...,M. This TSS model fulfills a coarse-to-fine focusing of driving anomaly. Actually, this structure is coincident with human cognition when finding anomaly from a video clip, and is presented as follows.

### 3.2. Temporally Abnormal Sub-Clip Detection

Temporal anomaly detection aims to extract the abnormal frames (frame-level anomaly). This is commonly fulfilled by frame-level consistency measurement of features or anomaly score, which does not consider the scene properties adequately. In driving scenarios, driving has a clear destination and path, and is manifestly a task-driven case. The investigations by [13,45] conclude that eye fixation of drivers is different corresponding to distinct semantic categories and reflects the driving context comprehensively. If an anomaly occurs, the eye fixation is destined to appear as a sudden change for the timely avoidance. Therefore, eye fixation correlates with driving anomaly directly. In this paper, we novelly introduce the top-down task-driven traffic saliency into the representation of the eye fixation, and design a simple but effective strategy to find the temporal anomaly. The work of [13] is employed as an attempt, which built a coarse-to-fine convolutional network on short sequences extracted from the DR(eye)VE dataset [45].

**Figure 3:**
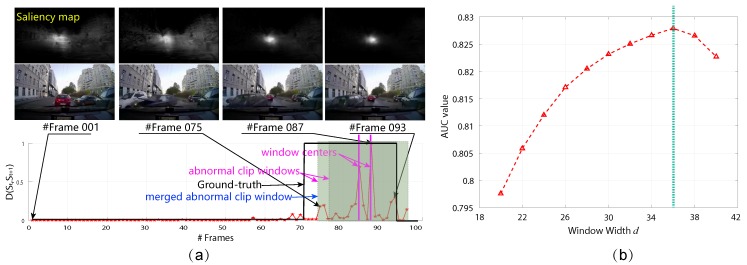
Illustration of temporally abnormal sub-clip detection. (**a**) a typical example; (**b**) the AUC value w.r.t. *d* when *K* is set as 4. We find that d=36 generates the best AUC value for training.

Specifically, assume the saliency maps at $t^{th}$ frame and ${\left(t+1\right)}^{th}$ frame are $S_{t}$ and $S_{t+1}$. Firstly, this work projects $S_{t}$ and $S_{t+1}$ in horizontal and vertical directions and generates the histograms of $\left\{s_{t}^{h},s_{t}^{v}\right\}$ and $\left\{s_{t+1}^{h},s_{t+1}^{v}\right\}$, respectively. Then, the difference $D(S_{t},S_{t+1})$ of $S_{t}$ and $S_{t+1}$ is defined as:

(1) $$D\left(S_{t},S_{t+1}\right)\;=\;\chi^{2}\left(s_{t}^{h},s_{t+1}^{h}\right)\cdot \chi^{2}\left(s_{t}^{v},s_{t+1}^{v}\right),$$

where $\chi^{2}\left(s_{t},s_{t+1}\right)=\frac{1}{2}\sum_{i=1}^{b}\frac{{\left|s_{t}\left(i\right)-s_{t+1}\left(i\right)\right|}^{2}}{s_{t}\left(i\right)+s_{t+1}\left(i\right)}$ is with *b* number of bins. After this computation for all of the frames in the given video clip, we normalize the difference into [0,1]. The between-frame computation is more perceptible to sudden change of fixation than incremental manners and easily partitions the clips. Taking Figure 3 as an example, the difference of saliency maps can reflect the temporal driving anomaly effectively. In order to contain most of the anomaly elements in the temporal module and adopt the role of traffic saliency, simultaneously, we set the frame windows centered as the frames with the top four largest $D(S_{t},S_{t+1})$ as temporal candidates ${\left\{C_{k}\right\}}_{k=1}^{K}$ for following detection and recounting procedure, where the window width is set as *d* frames. In this paper, we employ 40 video clips for training the best *d* and put this configuration into other clips. Note that, if the selected windows for the temporal candidates overlap, we merge them as one sub-clip, as illustrated in Figure 3.

### 3.3. Temporal-Spatially Local Anomaly Detection

After providing temporal candidates ${\left\{C_{k}\right\}}_{k=1}^{K}$, this paper further detects the local anomaly within them. In [46], anomaly is defined as suspicious and deviated observation from the others, so anomaly detection is generally formulated as finding the outlier deviating from the majority of observations. Within this field, isolation forest (iForest) [29] is a novel and unsupervised approach, which judges the anomaly by partitioning the samples with isolation trees (iTrees) and treats the samples with short average path lengths (APL) on the iTrees as anomaly. This is because it is easy for the anomaly to be partitioned earlier when iTree grows. Because of its efficiency and simplicity, we introduce it into this work while performing a new and effective ensemble.

**Figure 4:**
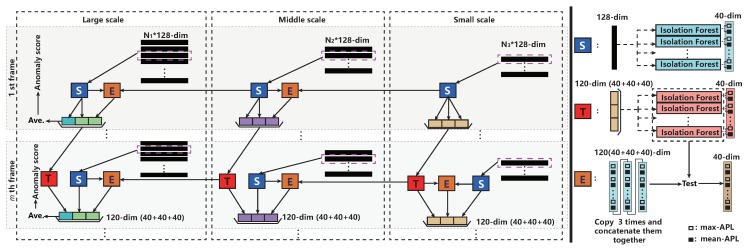
The flowchart of temporal-spatially local anomaly detection, wherein “**T**” represents the temporal iForest ensemble, “**S**” is the spatial iForest ensemble, and “**E**” specifies the module for testing the spatial features (obtained by “**S**” on the current scale) by “**T**” obtained by smaller scale. In the beginning, we only have the S module which detects the abnormal image part with an ensemble of multiple isolation forest in different scales. Then, the “**T**” module (also an ensemble of multiple isolation forest) aims to associate the detected spatial anomaly to subsequent frames, so as to obtain a consistent spatial anomaly results over different frames, and resist the error of optical flow. “**E**” module serves as a bridge over different scales, involving testing the results of “**T**” module from smaller scales by the “**S**” module on the current scale, which wants to leverage the supervision role of smaller scales to the current scale, and make a refined spatial anomaly detection.

To be specific, we propose a multi-scale temporal-spatial isolation forest (MSTS-iForest) to detect the local anomaly within each frame. The detailed flowchart is demonstrated in Figure 4. The advantages of this ensemble have two aspects: (1) making the obtained local anomalies consistent in temporal dimension; and (2) manifesting the local anomaly in spatial dimension. In this paper, we enforce the histogram of optical flow (HOF) to represent the image, and we claim that the efficient method contributed by Liu [47] is enough to evaluate. The detailed flowchart is explained as follows.

Multi-scale motion feature generation. This work adopts the HOF to represent the motion feature of images. Specifically, we resize the image into 220×220, and compute the optical flow field $\left[V_{x}\in R^{220\times 220},V_{y}\in R^{220\times 220}\right]$. For the multi-scale representation, we partition the motion field as non-overlapping 44×44, 20×20, and 10×10 blocks and calculate HOF of each block. Here, we set the bin number of HOF as 128. Consequently, we can obtain $N_{1}\times 128$-dim, $N_{2}\times 128$-dim, and $N_{3}\times 128$ feature matrixes, where $N_{1}$, $N_{2}$ and $N_{3}$ are 1936, 400, and 100, respectively, denoted in Figure 4.

Spatial iForest ensemble. For one sample, it is better to utilize multiple iForests to boost the accuracy of isolating process [29], i.e., obtaining accurate APL for each sample. Therefore, this work makes an ensemble of multiple iForests to spatially detect the local anomaly, and each forest has multiple iTrees, denoted by “S” module in Figure 4. We take the max-APL and mean-APL of each sample on each iForest to represent the anomaly degree, which is inspired by the fact that max-APL denotes the distribution representing the densest attribute of input samples, and mean-APL specifies the average distribution of attribute. Attribute means the values belonging to the range of the [minimum, maximum] of samples. This work sets the number of iForests as 20, and each iForest has 25 iTrees. Consequently, there is a 40-dim vector generated for one sample. Note that, in the “S” module, the input sample is the HOF feature vector with 128-dim.

Temporal iForest ensemble. In order to involve the problem of estimation error of optical flow and make the obtained local anomaly consistent and more obvious, this work designs the temporal module, denoted by “**T**” in Figure 4. This module further trains multiple iForests by anomaly distributions in the previous frame, and has a role of memory learning, by which the temporal anomaly consistency can be guaranteed, and make the local anomaly more obvious by temporal-spatially memory learning, which is validated as the key module in MSTS-iForest.

Multi-scale temporal-spatial ensemble. With the “S” module and “T” module in different scales, we ensemble them by bridging the “T” module on a smaller scale with “S” module on the current scale. This is because the smaller scale can play a supervision role for the larger scale because of the smoothness effect of the larger block. To be specific, we clone the 40-dim vector generated by “S” of current scale to triplet, and concatenate them together as 120-dim, then feed it into the “T” module on a smaller scale to generate a new 40-dim vector, denoted as “E” module. Incorporating with the “S”,“T” module, and the “E” module, we can generate a new 120-dim vector for representing the anomaly distribution of a new frame temporal-spatially learned. For the first frame, we only have the “S” module for each scale. Implementation details: The sub-sample size for training iForest is 256 for large and middle scales, and is set as 80 for the small scale because of the total sample number limitation. This configuration performs best in this work.

By the aforementioned modules, the anomaly score of each sample is generated by the “T” module in the highest scale, and computed by:

(2) $$s\left(HOF,N_{1}\right)=2^{-\frac{Ave(APL(HOF))}{c(N_{1})}},$$

where $c(N_{1})$ is average path length of unsuccessful search, HOF is the input sample, and $c\left(N_{1}\right)=2H\left(N_{1}-1\right)-\left(2\left(N_{1}-1\right)/N_{1}\right)$, where $N_{1}$ is the sample number of the large scale, i.e., 1936 in this work, and H(i) is harmonic number and estimated by ln(i)+0.5772156649 (Euler’s constant), which is borrowed from [29]. Ave(APL(HOF)) denotes the average APL of HOF on all of the iForests in “T”. After obtaining the anomaly score for each sample, we reshape the sample matrix back into a 44×44 grid, and enlarge it into original image size, i.e., 220×220, by bilinear interpolation, which is denoted as the anomaly map, wherein the anomaly regions are denoted as $A_{m}^{k}$ for a frame $X_{m}^{k}$ in $C_{k}$.

### 3.4. Temporal-Spatial-Semantical Anomaly Recounting

As for the final temporal-spatial-semantic module for driving anomaly analysis, this work novelly recounts the causal relationship between anomaly and potential causes, which is different from the direct perceiving for object, attribute, and action learning [44]. This work gives an unsupervised reasoning by learning perceptual causality, which is inspired by the work [48] contributed by Fire and Zhu. They learned the perceptual causality of “actions” and “effect” by electing the most informative causal relations sequentially in terms of maximizing the information gain. In this paper, the “effect” is represented as the anomaly regions. As for the “action”, we denote them as the semantic variation within the anomaly regions. For example, for an anomaly of “car hits person”, “car changed into person”, “person changed into road”, and “car changed into road” can potentially make a useful representation. Even so, the accurate learning of the most informative semantic variation in the anomaly regions remains challenging. The difficulties are mainly: (1) the accurate traffic element segmentation of an image; and (2) the robust perceptual causality learning. In addition, the rarity of anomaly makes the training frameworks (e.g., RNNs and CNNs) rather difficult for causality analysis.

Specifically, we introduce the fully convolutional networks (FCN) utilizing VGG-16 [15] fine-tuned by the Cityscapes dataset [16] to segment each frame $X_{m}^{k}\in C_{k}$, m=1,...,M, k=1,...,K into $F_{m}^{k}$. Then, we prepare the basic units of traffic element variation in frame $X_{m}$ by collecting the semantic variation of pixels within $A_{m}^{k}$ and $A_{m-1}^{k}$ corresponding to $F_{m-1}^{k}$ and $F_{m}^{k}$. With the obtained basic units, this paper learns the perceptual causality between them with the anomaly by measuring the temporal-spatial co-occurrence and the information gain of each unit. Suppose there are *W* basic units ($W=C^{2}$, where *C* = 30 is the traffic element classes in Cityscapes dataset) representing different semantic variations. We calculate their co-occurrence in spatial dimension by counting the frequency $f_{m-1}$ = $\left\{f_{m-1}^{1},f_{m-1}^{2},...,f_{m-1}^{W}\right\}$ relatively to all the basic unit number, which is fed into the historical co-occurrence distribution, defined as:

(3) $$H_{m}=\alpha H_{m-1}+\left(1-\alpha \right)f_{m-1},$$

where $H\in R^{1\times C^{2}}$ is the temporal-spatial co-occurrence distribution of all kinds of semantic variation, each bin of H correlates to a semantic variation, and α is the learning rate set as 0.3 in all experiments. Based on [48], the most informative causal relation is defined as the one with the max information gain. This is referential to this work because anomaly usually causes a sudden change of distribution of spatial co-occurrence. Hence, we treat $H_{m}$ as the current model, and evaluate the information gain of $f_{m}^{i}$ in newly observed data $f_{m}$ to $H_{m}$ by computing the ratio of Kullback–Leibler (KL) divergences with and without $f_{m}^{i}$:

(4) $$\overset{⌢}{f}=\arg\max_{f_{m}^{i}}\frac{\mathrm{KL}(H_{m-1}||f_{m})}{\mathrm{KL}(H_{m-1}||f_{m}/f_{m}^{i})},$$

where KL(·||·) is the KL divergence, and $\overset{⌢}{f}$ is the elected most informative causal relation in the *t*th frame. To recount the anomaly regions in each frame, we select the semantic variations with top three $\overset{⌢}{f}$. Then, we aggregate the primary semantic variations in each frame and treat the ones with top three frequency over all frames as the clip recounting.

## 4. Experiments and Analysis

### 4.1. Dataset

Based on the investigation, there is no publicly available dataset for validating the proposed method requiring a temporal-spatial-semantical labeling. The most related one is the crowd-sourced dashcam video dataset for accident anticipation (http://aliensunmin.github.io/project/dashcam/) contributed by [32], which only labeled the temporal occasion of the accident. Actually, the anomaly may appear earlier than the accident. Therefore, this paper constructs a new driving anomaly video dataset (Drive-Anomaly106, https://github.com/ZHU912010/Driving-Anomaly-Detection) containing 106 video clips (each one has 100 frames), which are temporal-spatial-semantically labeled carefully by ourselves, some of which are collected from [32]. The resolution of the frame in the clips are 1280×720 or 476×265, which are normalized to 360×200. The anomaly regions are masked by their instance-level contours. For anomaly labeling, we recommended two principles: (1) The anomaly is object-oriented and threatening to the ego-vehicle, such as vehicle crossing, overtaking, and so on; (2) The anomaly owns a manifest trend to cause an accident with ego-vehicle or other objects.

**Figure 5:**
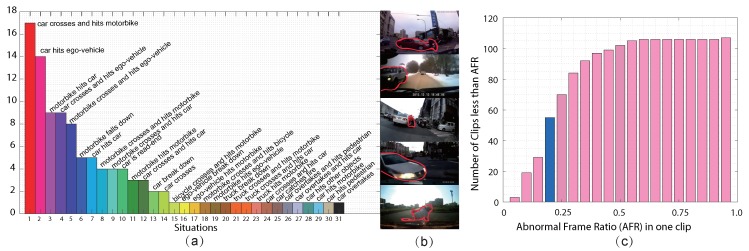
The statistics of Drive-Anomaly106. (**a**) the situation distribution; (**b**) the typical frames in the top five kinds of situations, where the anomaly regions are marked by red contour; (**c**) the abnormal frame ratio of the clips in Drive-Anomaly106.

Figure 5a demonstrates the distribution of driving anomaly situations, and typical frames in the top five kinds are successively shown. From these statistics, the driving anomaly may exhibit various forms and the difference of some situations is ambiguous, such as “car crosses and hits ego-vehicle” and “car hits ego-vehicle” because of the complicated motion condition. In addition, we also show the abnormal frame ratio (AFR) statistics for all the clips in Figure 5c. It can be observed that there are almost half of the clips whose AFRs are larger than 20%, which to some extent violates the temporal rarity of anomaly, e.g., [4]. In addition, distinct weather and light conditions further strengthen the challenge. Drive-Anomaly106 is the first large-scale driving anomaly dataset fully labeled as far as we know, and will be released in the near future.

### 4.2. Implementation Details

For proving the effectiveness of this work, this paper firstly compares the detection performance on different components in this work and with state-of-the-art unsupervised anomaly detectors. Then, we give an analysis for the driving anomaly recounting.

Comparison of different components for detection. Since this work proposes a progressive temporal-spatial-semantic analysis framework, this paper will evaluate the detection performance in each module. In order to examine the ability for finding the abnormal frames, Precision and Recall of the detected abnormal frame are employed, where $Precision=\;\frac{TP}{TP\;+\;FN}$ and $Recall=\;\frac{TP}{TP\;+\;FP}$, where TP, FP, and FN specify the numbers of truly detected abnormal frames, undetected abnormal frames and wrongly detected abnormal frames, respectively. Albeit some other metrics, e.g., ROC and AUC, commonly adopted in existing anomaly detection methods, can also be used for evaluation, Recall value in this work is treated as the index for qualifying the temporal anomaly candidates because we want all of the abnormal frames in each video clip to be able to be fed into the following modules. In terms of temporal-spatial modules, this paper employs the standard pixel-level ROC and AUC metrics to evaluate the performance.

Detection comparison with the state-of-the-art. Because most of the anomaly detectors are supervised modules, this paper validates the superior performance with other unsupervised anomaly detectors, viz., basic iForest [29], one-class-SVM (OC-SVM) [49], and robust deep auto-encoder (RDA) [50]. The entire pipeline, putting all frames in each clip into evaluation, is compared here. In addition, for driving anomaly detection, we also compare our method with the incremental graph regularized least soft-threshold squares (iGRLSS), which is used for the motion consistency measurement in the work of [37] for driving scenarios. The detailed implementations for these methods are:

(1) iForest: We perform the iForest spatially on the large scale channel, with the same configuration for number of iForests, iTrees, and partitioning blocks;
(2) OC-SVM: The partitioned optical flow field in each frame is used to train the boundaries with an RBF kernel, and the anomaly score of each sample is determined by the distance to the decision boundary;
(3) RDA: RDA aims to find the principal component with a detection of outlier using a multi-layer structure. This work introduces the $\ell_{2,1}$ penalty, and compared several parameter combinations and used parameters that performed best (λ = 0.00056 and layer size is 128, 80, and 100). The anomaly is determined by checking the reconstruction error of the partitioned 44×44 blocks of optical flow field, where the instance dimension (HOF feature) is 128, the same as the proposed method;
(4) iGRLSS: Strictly speaking, iGRLSS is a weak-supervised method, which first segmented the frame into many superpixels, and treated the first 10 frames of each clips as normal. Then, the temporal consistency with the pre-defined normal patterns in each clip was examined. This paper sets the superpixel number as 125 following its setting of [37], and adopts five frames to update the dictionaries in iGRLSS.

For comparing the performance, this work focuses on the standard pixel-level ROC and AUC values to evaluate because of its more attractive attention for boosting. These methods can be treated as the state-of-the-art for unsupervised anomaly detection.

### 4.3. Evaluation on Different Detection Components

**Figure 6:**
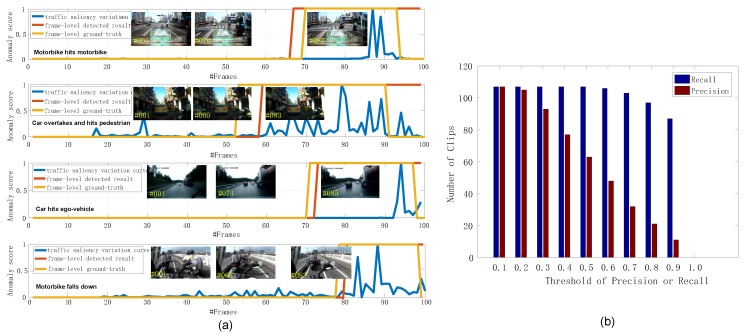
Evaluation on temporal module. (**a**) are typical clips demonstrating the frame-level anomaly results and (**b**) presents the number of clips obtained larger precision and recall value than a pre-defined threshold.

Evaluation on temporal abnormal sub-clips. For the temporal module, we employ the qualitative and quantitative evaluations, wherein qualitative evaluation is provided by demonstrating the anomaly curves of some typical video clips, and the number of clips obtaining larger precision and recall value than a pre-defined threshold is utilized for quantitative evaluation. The detailed results are shown in Figure 6, where the beginning frame of driving anomaly can be localized effectively, such as the 70th frame, 60th frame and 74th frame in the clips in Figure 6a, and the anomaly frames can be contained to a large extent for these clips. Actually, this phenomenon is universal for most of the clips, and almost all of the abnormal sub-clips can be localized by the temporal module, proved by the consistent Recall value in Figure 6b under different thresholds. In other words, the temporal module can remove a large proportion of frames while recalling the anomaly frames. From this figure, we can observe that the traffic saliency can provide promising guidance for temporal anomaly.

**Figure 7:**
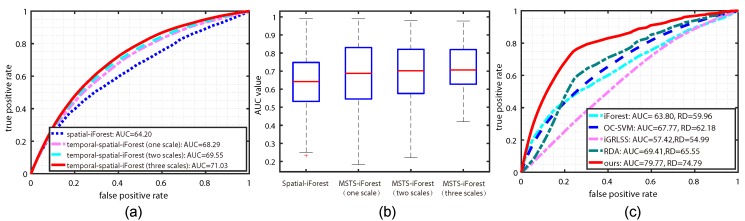
The performance comparison. (**a**) represents the pixel-level ROC curves and the average AUC values (%) of iForest and MSTS-iForest, (**b**) denotes the dispersion degree of the AUC values on all the clips, and (**c**) is the pixel-level ROC curves of various anomaly detectors.

Evaluation on temporal-spatial pixel-level anomaly. In terms of the temporal-spatial module, this work proposes a multi-scale temporal-spatial isolation forest (MSTS-iForest) to detect the local anomaly within each frame. Therefore, the role of each component in MSTS-iForest is evaluated in this subsection. In this module, we employ the standard pixel-level ROC and area under ROC (AUC) to quantitatively qualify the performance. Note that the evaluation on this part builds on the obtained temporally abnormal sub-clips for a fair and clear comparison. The results are demonstrated in Figure 7a,b. From these sub-figures, we can see that the basic spatial iForest is manifestly poorer than MSTS-iForest (three scales) (having 6.83% gap). The reason behind this is the key role of temporal learning, which can memorize the historic normal situations and make the anomaly more obvious (proven in Section 4.4, giving a better and concise comparison with other methods). In addition, optical flow usually generates estimation error, and the temporal module can restrict it by a consistency consideration. With the temporal module, the performance is largely boosted while demonstrating a similar average AUC for different scale numbers. However, from Figure 7b, one scale configuration generates more dispersive AUC values for all the clips and many clips obtain poor AUC. In other words, the multi-scale form is more robust than the single scale. Actually, this phenomenon is caused by the fact that the anomaly regions in multi-scale form are more obvious than the ones on a lesser scale because of the supervision role of the larger blocks in a smaller scale channel.

### 4.4. Comparison with the State-Of-The-Art

**Figure 8:**
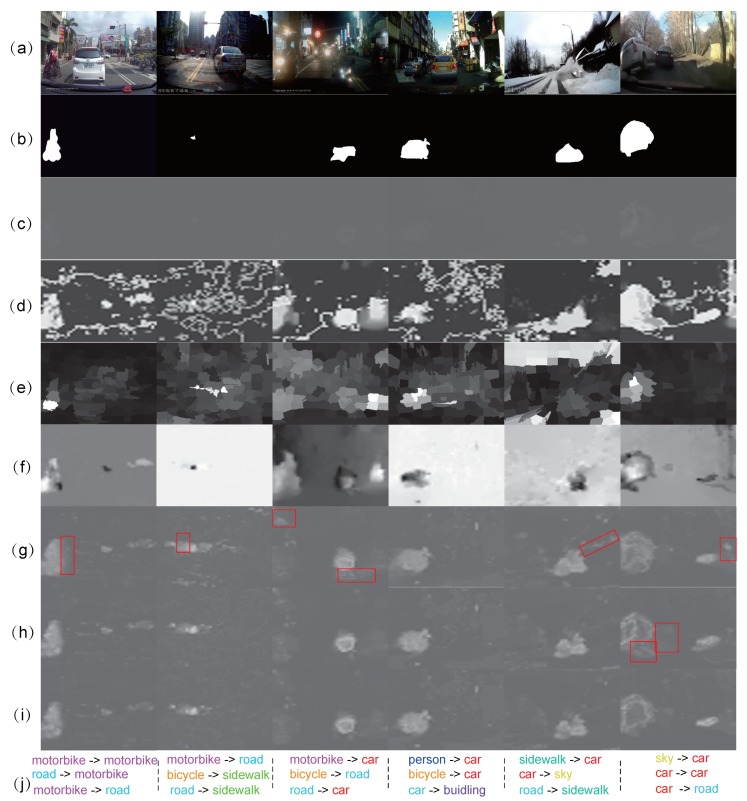
Typical examples for demonstrating the anomaly map by different detectors. (**a**) original frames; (**b**) ground-truth of anomaly regions; (**c**) denotes the results of basic iForest [29]; (**d**–**f**) represent the results of OC-SVM [49], iGRLSS [37], and RDA [50], respectively. The results of our method are presented by (**g**) our method with only one scale, (**h**) with two scales, and (**i**) with three scales; (**j**) denotes the recounting results of the related clips.

In this comparison, we evaluate the entire pipeline of our method with state-of-the-art unsupervised approaches. To be specific, we treat frames beyond the detected abnormal sub-clips as normal, and run the other methods on the entire clip. The performance of them are compared by the pixel-level ROC curves, area under ROC curve (AUC), and the ratio of detection (RD), as was explained in Li et al. [6]. These metrics are based on the pixel-level anomaly map and the ground-truth. The detailed comparison results are quantitatively shown in Figure 7c, and qualitatively demonstrated in Figure 8.

Figure 7c shows that the proposed method is apparently superior to other ones, and RDA and iForest obtain the second and third rank. For this observation, the behind reasons are summarized as: (1) a large proportion of frames in one clip are removed by the temporal module, therefore reducing a large percentage of false positives; (2) OC-SVM, RDA, and iForest do not have the temporal consideration for anomaly measurement, which is actually vulnerable to estimation error of optical flow and other disturbing regions; (3) iGRLSS only considers the temporal consistency. Albeit it maintains the spatial objectness, the spatial anomaly is not involved. Figure 8 demonstrates some typical frames for an in-depth comparison. From this figure, we can observe that iForest cannot manifest the boundary of anomaly and normal pattern clearly, and generates an unobservable anomaly map. OC-SVM highlights many irrelevant points as anomalies, which may be caused by the inaccurate boundary learned for separation because OC-SVM may learn multiple boundaries for samples with multi-modal distribution. As for the RDA, it finds the anomaly with large reconstruction error to original data; while it might be disturbed by the multi-modal distribution of the optical field, reversed determination emerges sometimes. With respect to our methods with different scale number, we can see that our method can obtain a relatively similar result. However, taking a deep observation, we find that more scales will weaken more background clutter, and generate clearer shapes complying with the ground-truth. The single scale easily generates “trailing smear” phenomena, marked by the red boxes in Figure 8. This is because, without the supervision of smaller scales, these points are also treated as the anomaly.

It is worth noting that the Drive-Anomaly106 dataset contains 16 video clips of nocturne driving, 22 video clips of heavy traffic, and 20 video clips of bad weather conditions, accounting for 15.09%, 20.75%, and 18.87% of the total, respectively. The proposed model still achieves good performance on the three special conditions. The AUC values of them achieve 83.24%, 79.10%, and 82.25%, as listed in Table 1, which even outperforms the overall level of 79.77%. It demonstrates that the proposed framework can ensure the effectiveness and robustness in different conditions.

### 4.5. Evaluation on Driving Anomaly Recounting

With respect to the driving anomaly recounting, we qualitatively demonstrate the top three causal relations for typical frames, as shown in Figure 8j. It is worth noting that the accuracy of recounting is quantized by measuring the number of clips that obtain a matched semantic variation for the anomaly regions. For example, for a “car hits motorbike” anomaly, if the obtained top three semantic variations have “car changed into motorbike” or “motorbike changed into car”, we treated this as an accurate recounting. Under the configuration in this work, we obtain 23 (relative to 106) clips accurately recounted. Actually, the performance of recounting binds closely to the semantic segmentation methods, e.g., FCN [15]. Therefore, the provided framework for driving anomaly recounting will be updated in the future.

### 4.6. Discussion

**Figure 9:**
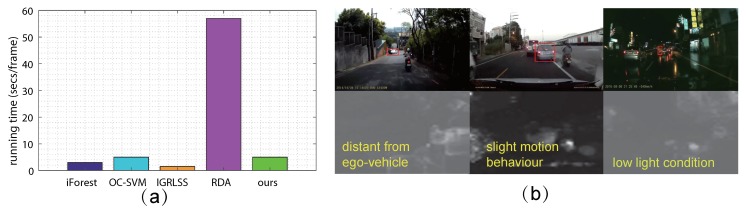
Discussion of for efficiency and failure situations. (**a**) demonstrates the efficiency comparison; and (**b**) shows the situations of anomaly distant from ego-vehicle, slight motion behavior, and imperceptible anomaly with low light condition, coupled with the detection results by ours.

Efficiency comparison. Although the proposed TSS model has three modules, the main time cost belongs to MSTS-iForest, depending on the iTree number *t*, sub-sample size β and instance number *n*. Based on the analysis in [29], the time complexities are O(tβlogβ) for training and O(ntlogβ). When we set t=500, β=256, n=1936 for the large scale channel, n=400 for the middle scale and n=100 for the small scale, incorporating the time cost for optical flow estimation, total running time for each frame is five seconds in average, and reduces to 4.3 s and 3.5 s with the decreasing of scale number, which is a little longer than three seconds only with spatial iForest for each frame, on the same PC platform with 2.70 GHz i7 CPU and 32 GB RAM. Therefore, the MSTS-iForest is an efficient but effective ensemble. In addition, we also compare our method with other approaches. The results are shown in Figure 9a. From this table, we can observe that iGRLSS is the most efficient one (1.5 s/frame), while it is poorer than other ones. RDA is the slowest one (57 s/frame) owing to the exhaustive training process. Our method shows a competitive efficiency but the best detection ability.

Failure situation analysis. Actually, this work tackles the driving anomaly with a large-scale dataset for the first time. The diversity of the anomaly situations makes the detection rather challenging, and inevitably encounters some failures. To summarize, the failures mainly appear in three kinds of circumstances: (1) anomaly distance from ego-vehicle, (2) anomaly with slight motion behavior, and (3) imperceptible anomaly in low light conditions. Some examples are shown in Figure 9b, marked by red boxes. These situations make the anomaly motion be rather unobservable, along with many disturbing and similar appearances. In the meantime, the motion in other normal regions may cover up the imperceptible motion change of anomalies. Therefore, camera motion compensation may be needed in the future.

## 5. Conclusions

This work addressed the driving anomaly detection and recounting problem by a progressive temporal-spatial-semantic analysis framework. This framework novelly incorporated the property of driving scenarios, and introduced a top-down traffic saliency relating to eye fixation of drivers to temporally find the sudden scene variation, likely the existing driving anomaly. Within the temporal candidate for driving anomaly found, this paper further examined the spatial anomaly region by a novel multi-scale temporal-spatial iForest (MSTS-iForest) that has a temporal memory learning and spatial anomaly highlighting ability for local anomaly detection. The driving anomaly recounting was exploited by a temporal-spatial-semantic perceptual learning, which adequately explored the temporal-spatial co-occurrence of semantic variation of anomaly regions. Exhaustive experiments demonstrated the superiority of the proposed framework.

## Figures and Tables

**Table 1:** The performance on the three special conditions, involving the scenarios with nocturne driving, heavy traffic and bad weather.

Conditions	Number of Sequences	Proportion (%)	AUC (%)
Nocturne Driving	16	15.09	83.24
Heavy Traffic	22	20.75	79.10
Bad Weather	20	18.87	82.25

## Supplementary Materials

Supplementary File 1
